# Effect of MRI acquisition acceleration via compressed sensing and parallel imaging on brain volumetry

**DOI:** 10.1007/s10334-020-00906-9

**Published:** 2021-01-27

**Authors:** Michael Dieckmeyer, Abhijit Guha Roy, Jyotirmay Senapati, Christian Wachinger, Lioba Grundl, Jörg Döpfert, Pere Ferrera Bertran, Andreas Lemke, Claus Zimmer, Jan S. Kirschke, Dennis M. Hedderich

**Affiliations:** 1 Department of Diagnostic and Interventional Neuroradiology, Klinikum Rechts Der Isar der Technischen Universität München, Ismaninger Str. 22, 81675 Munich, Germany; 2 Lab for Artificial Intelligence in Medical Imaging, Klinik und Poliklinik Für Kinder- und Jugendpsychiatrie, Psychosomatik und Psychotherapie, Klinikum der Universität München, LMU München, Waltherstr. 23, 80337 Munich, Germany; 3 mediaire GmbH, Möckernstraße 63, 10965 Berlin, Germany

**Keywords:** Brain, Magnetic resonance imaging, Acceleration, Systematic bias

## Abstract

**Objectives:**

To investigate the effect of compressed SENSE (CS), an acceleration technique combining parallel imaging and compressed sensing, on potential bias and precision of brain volumetry and evaluate it in the context of normative brain volumetry.

**Materials and methods:**

In total, 171 scans from scan-rescan experiments on three healthy subjects were analyzed. Each subject received 3D-T1-weighted brain MRI scans at increasing degrees of acceleration (CS-factor = 1/4/8/12/16/20/32). Single-scan acquisition times ranged from 00:41 min (CS-factor = 32) to 21:52 min (CS-factor = 1). Brain segmentation and volumetry was performed using two different software tools: *md.brain*, a proprietary software based on voxel-based morphometry, and *FreeSurfer*, an open-source software based on surface-based morphometry. Four sub-volumes were analyzed: brain parenchyma (BP), total gray matter, total white matter, and cerebrospinal fluid (CSF). Coefficient of variation (CoV) of the repeated measurements as a measure of intra-subject reliability was calculated. Intraclass correlation coefficient (ICC) with regard to increasing CS-factor was calculated as another measure of reliability. Noise-to-contrast ratio as a measure of image quality was calculated for each dataset to analyze the association between acceleration factor, noise and volumetric brain measurements.

**Results:**

For all sub-volumes, there is a systematic bias proportional to the CS-factor which is dependent on the utilized software and subvolume. Measured volumes deviated significantly from the reference standard (CS-factor = 1), e.g. ranging from 1 to 13% for BP. The CS-induced systematic bias is driven by increased image noise. Except for CSF, reliability of brain volumetry remains high, demonstrated by low CoV (< 1% for CS-factor up to 20) and good to excellent ICC for CS-factor up to 12.

**Conclusion:**

CS-acceleration has a systematic biasing effect on volumetric brain measurements.

**Supplementary Information:**

The online version contains supplementary material available at 10.1007/s10334-020-00906-9.

## Introduction

Thanks to technical advances in the recent years, whole-brain segmentation and volumetry can be performed fully automated and MRI-based brain volumetry is increasingly used in the clinical setting [1].

Its application has great potential to support disease diagnosis, improve the understanding of pathomechanisms, track disease progression and monitor treatment effects [2]. In addition to mere visual image evaluation, it has the potential to deliver a more precise and objective evaluation of patients with neurodegenerative diseases in clinical practice. One important application is normative brain volumetry (NBV), which compares measured volumes of different brain structures to an age- and gender-adjusted healthy cohort to reveal deviations from normal volumes. Recently, NBV has been shown to improve diagnostic accuracy of neurodegenerative atrophy patterns as well as interrater reliability for detection and differential diagnosis of neurodegenerative diseases [3–6]. It is of note that brain volumetry has a far broader range of applications, such as in the investigation of brain development [7].

Whole brain volumetry is usually based on high-quality three-dimensional T1-weighted magnetization-prepared rapid gradient-echo sequences (MPRAGE) [8]. The necessity to encode a large number of k-space lines as well as the preceding inversion time (TI), necessary for the T1-weighted contrast, results in long scan times at low acceleration factors. This might be problematic for widespread clinical adoption considering the increasing workloads and notoriously tight schedules in radiological departments and practices [9]. The long scan times also increase the susceptibility for motion artifacts leading to reduced image quality and accuracy of brain volumetry.

Recent advances on the acquisition side, such as compressed SENSE (CS), GRAPPA or Wave-CAIPI, aim at significantly reducing scan times [10–13]. CS represents a combination of the parallel imaging (PI) technique SENSE [14] and compressed sensing [15, 16]. Through the application of sparsity constraints and k-space undersampling, a substantial image acquisition acceleration can be achieved. It was recently shown that the implementation of CS into clinical practice of neuroimaging leads to decreased scan times at preserved visual image quality [17, 18]. Volumetric brain MRI using ultrafast magnetization-prepared rapid gradient-echo (MP-RAGE) sequences has been evaluated recently. The authors used Wave-Controlled Aliasing in Parallel Imaging (Wave-CAIPI; Siemens) for acquisition acceleration and reported low intra-individual variability as well as comparable morphometric estimates between accelerated and non-accelerated scans [19, 20].

However, it is unknown whether accelerated acquisition through CS impacts whole-brain volumetric measurements. Therefore, the aim of our study was to evaluate the effect of CS on brain volumetry and objective image quality using well-controlled test–retest data from three healthy individuals. Two brain volumetry tools were applied to cover distinct morphometry approaches to evaluate the influence of CS on measured brain volumes in surface-based morphometry and voxel-based morphometry.

## Materials and methods

### Study subjects

Three healthy subjects (1 female, 2 males; age 27–31 years) were recruited for this study and gave informed written consent. The study was approved by the local institutional review board (IRB). Each subject underwent three consecutive MRI scans of the brain on different days within a seven-day period. To minimize changes in brain volume depending on the time of day, all scans were performed between 06:00 a.m. and 08:00 a.m. Each MRI scan followed the same protocol as specified below.

### MRI protocol

Imaging was performed on a 3 T MRI scanner (Philips Ingenia, Philips, Best, The Netherlands) using a 32-channel head coil.

CS with increasing degrees of k-space undersampling was used for image acquisition acceleration. The degree of acceleration is expressed by the compressed SENSE reduction factor (CS-factor). Each imaging dataset was inspected visually by a radiologist to assure the absence of severe motion artifacts.

The MRI protocol consisted of a three-dimensional T1-weighted turbo field echo sequence (3D-T1w-TFE) which was performed at increasing CS-factors of 1 (no acceleration), 4, 8, 12, 16, 20 and 32. For CS-factor = 1, the sequence was performed once per scan date, resulting in a total of three identical measurements per subject. For each CS-factor ≥ 4, the sequence was performed three times per scan date, resulting in a total of nine identical measurements per subject. Thus, in total, 171 3D-T1w datasets were acquired.

The acquisition times for each CS-factor are displayed in Table 1. The total exam time per scan date was 62 min. The other sequence parameters were as follows: acquired voxel size, 1 × 1 × 1 mm^3^; field of view, 250 mm × 250 mm × 180 mm (feet-head × anterior–posterior × right-left; FH × AP × RL); TR, 600 ms; TE = 28 ms; acquisition plane, sagittal.

**Table 1:** Acquisition times (in minutes and seconds) of the applied 3D-T1w-TFE sequence for each CS-factor

CS-factor	1	4	8	12	16	20	32
Acquisition time	21:52	05:18	02:41	01:49	01:22	01:05	00:41

*CS* compressed SENSE

### Image and data analysis

Based on the acquired 3D-T1w-TFE datasets, cortical reconstruction and volumetric segmentation was performed with two different processing tools:

(i) The commercially available and CE-certified software-tool md.brain v1.1.1 (mediaire GmbH, Berlin, Germany). The software performs a segmentation of different brain regions based on voxel-based morphometry (VBM) and statistical parametric mapping (SPM) and then determines their volumes [21–24]. Throughout the remainder of this article, this processing tool will be referred to as MDB.
(ii) FreeSurfer image analysis suite v6.0, which is documented and freely available for download online [25, 26]. The software performs a segmentation of different brain regions based on surface-based morphometry according to Fischl et al. [27, 28]. Seven of the 27 datasets that were acquired at CS-factor = 32 could not be analyzed with FS due to insufficient image quality. The most likely reason is the failed convergence of the internal optimization algorithm of the FS software that uses a set of high-quality reference MRI volumes. Throughout the remainder of this article, this processing tool will be referred to as FS.

The following sub-volumes were analyzed: brain parenchyma (equal to combined total gray and white matter volume; BP), total gray matter (GM), total white matter (WM), and cerebrospinal fluid (CSF).

Mean and standard deviation (SD) of the repeated measurements were calculated for each CS-factor and subvolume. The coefficient of variation (CoV) as a measure of intra-subject reliability was calculated as CoV = SD/mean.

Intra-class correlation (ICC) was calculated as another measure of reliability with respect to CS-factor. Because of the presence of systematic bias, we chose ICC(C,1) according to the McGraw and Wong notation [29] as the appropriate type of ICC [30]. For each software package and each of the four analyzed brain volumes (BP, WM, GM and CSF), ICC was calculated pairwise to assess reliability between measurements at CS = 1 and CS = 4, 8, 12, 16, 20 and 32, respectively, using the following notation: ICC_1,4_, ICC_1,8_, etc.

The linear relationship between the applied CS-factor and the measured brain volume was assessed by calculating Pearson correlation coefficient R for both software tools and each subvolume.

Noise-to-contrast ratio (NCR) as a measure of image quality was calculated for each acquired dataset using the CAT12 toolbox by measuring the local standard deviation in the optimized WM segment scaled by the minimum tissue contrast [31].

To assess within-session and across-session reproducibility and the potential averaging effects of within-session data, for each CS-factor > 1, we calculated mean and SD within a single session (3 repeated scans per session) and across sessions (mean of each session for 3 sessions), respectively.

### Statistical analysis

Mean volumes calculated for different CS-factors were compared using paired two-sided *t* tests with Bonferroni correction to address multiple comparisons. Cohen’s *d* as measure of effect size was calculated for significant values of relevant comparisons.

Outliers of the volume measures were detected using the Grubbs test and removed [32]. All statistical tests were performed at a significance level *α* = 0.05. Statistical analyses were performed using MATLAB (The MathWorks, Natick, USA).

## Results

### Measured brain volumes

**Fig. 1:**
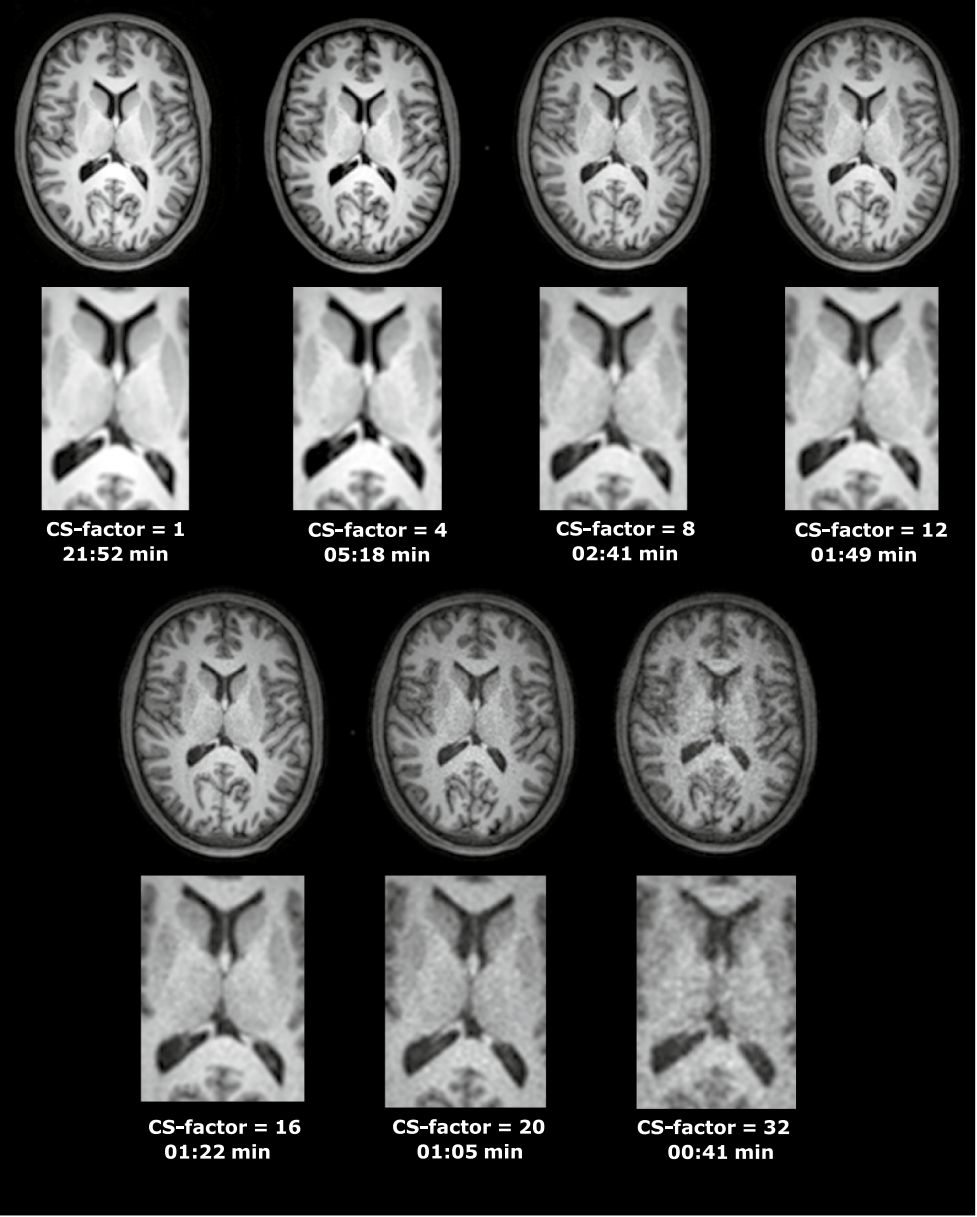
Sample axial reconstructions of the acquired 3D-T1-weighted brain images at increasing CS-factors of one of the subjects (enlarged view of the image center below each full FOV image). *CS* compressed SENSE

Representative axial brain images of WM, GM and CSF at increasing CS-factors of one of the subjects are shown in Fig. 1. Supplementary Figures 2a (MDB) and 2b (FS) display representative axial and coronal segmentation overlays of WM, GM and CSF at increasing CS-factors of one of the subjects. From this figure, it can be appreciated visually how the increasing CS-factor affects the segmentations at the interfaces of the three compartments, in particular for CS-factors ≥ 20.

**Fig. 2:**
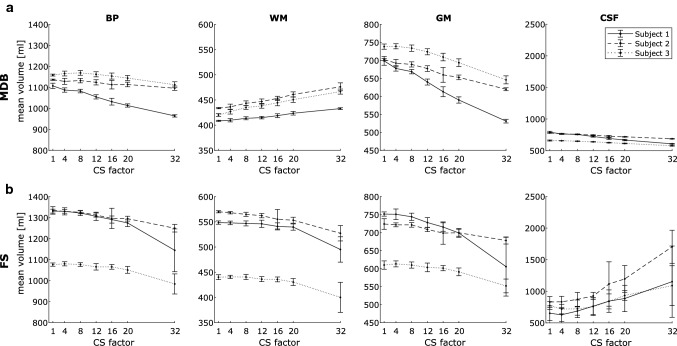
MDB-based (**a**, upper row) and FS-based (**b**, lower row) mean ± 2SD of absolute segmented volumes of the three examined subjects as a function of CS-factor. *CS* compressed SENSE, *MDB* md.brain segmentation and volumetry tool, *FS* FreeSurfer segmentation and volumetry tool, *BP* brain parenchyma, *WM* white matter, *GM* gray matter, *CSF* cerebrospinal fluid, *SD* standard deviation

**Fig. 3:**
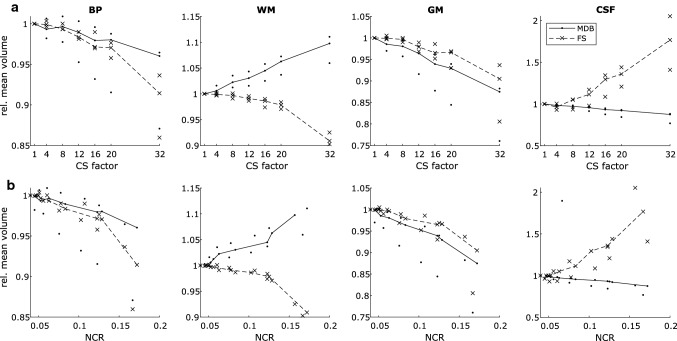
Segmented volumes relative to the volume at CS-factor = 1, as a function of CS-factor (**a**, upper row) and NCR (**b**, lower row). Each subject is represented by a dot (MDB) or cross (FS) and the median across the three subjects is represented by a solid (MDB) or dashed (FS) line. *NCR* noise-to-contrast ratio, *MDB* md.brain segmentation and volumetry tool, *FS* FreeSurfer segmentation and volumetry tool, *BP* brain parenchyma, *WM* white matter, *GM* gray matter, *CSF* cerebrospinal fluid, *CS* compressed SENSE

Mean and standard deviation of the absolute volumes were calculated for MDB (Fig. 2a) and FS (Fig. 2b). The analyzed volumes are displayed as a function of the employed CS-factor for each of the three subjects. Calculated volumes relative to the volume at CS-factor = 1 are shown as mean across all subjects in Fig. 3a.

For all analyzed sub-volumes and each subject, there is a systematic bias proportional to the employed CS-factor. Degree and direction of the bias are dependent on the utilized processing tool as well as the analyzed sub-volume.

#### MDB-based volumetry

For WM, the calculated volumes increase with increasing CS-factor. For all other sub-volumes, the calculated volumes decrease with increasing CS-factor (Fig. 3a). Combining the measured relative volumes of all subjects, the observed differences between consecutive CS-factors (CS-factor = 1 vs. 4, 4 vs. 8, etc.) were significant (*p* < 0.05) for all subvolumes except for the difference between CS-factors 4 and 8 in BP (Tables 2, 3). The effect size assessed by Cohen’s *d* was larger for MDB than for FS (Tables 3, 5).

**Table 2:** MDB-based volumes relative to CS-factor = 1, mean and SD across all subjects

CS-factor	4		8		12		16		20		32	
	Mean	SD	Mean	SD	Mean	SD	Mean	SD	Mean	SD	Mean	SD
BP	0.9939	0.0114	0.9944	0.0139	0.9817	0.0223	0.9691	0.0286	0.9611	0.0334	0.9318	0.0443
GM	1.0082	0.0077	1.0234	0.0105	1.0297	0.0125	1.0426	0.0148	1.0573	0.0162	1.0892	0.0229
WM	0.9855	0.0142	0.9773	0.0164	0.9534	0.0285	0.9257	0.0375	0.9044	0.0438	0.8391	0.0571
CSF	0.9811	0.0125	0.9703	0.0121	0.9463	0.0239	0.9190	0.0328	0.8981	0.0398	0.8414	0.0532

Relative volumes were calculated individually for each subject

*CS* compressed SENSE, *SD* standard deviation, *MDB* md.brain segmentation and volumetry tool, *BP* brain parenchyma, *WM* white matter, *GM* gray matter, *CSF* cerebrospinal fluid

**Table 3:** Comparison between MDB-based relative volumes between consecutive CS-factors (1 vs. 4, 4 vs. 8, etc.) across all subjects: *p*-values of paired *t* tests, Cohen’s *d* as measure of effect size

CS-factors	1 vs. 4		4 vs. 8		8 vs. 12		12 vs. 16		16 vs. 20		20 vs. 32	
	*p*	*d*	*p*	*d*	*p*	*d*	*p*	*d*	*p*	*d*	*p*	*d*
BP	0.010	0.53	0.591	–	< 0.001*	1.28	< 0.001*	1.38	0.001	0.75	< 0.001*	2.33
GM	< 0.001*	− 1.07	< 0.001*	− 2.99	< 0.001*	− 1.14	< 0.001*	− 2.54	< 0.001*	− 2.18	< 0.001*	− 3.88
WM	< 0.001*	1.03	< 0.001*	1.75	< 0.001*	1.76	< 0.001*	2.12	< 0.001*	1.45	< 0.001*	4.03
CSF	< 0.001*	1.51	< 0.001*	3.14	< 0.001*	1.82	< 0.001*	2.56	< 0.001*	1.77	< 0.001*	3.61

*CS* compressed SENSE, *MDB* md.brain segmentation and volumetry tool, *BP* brain parenchyma, *WM* white matter, *GM* gray matter, *CSF* cerebrospinal fluid

*Marks significant difference to volume at consecutive CS-factor (*p* < 0.05)

#### FS-based volumetry

For CSF, the calculated volumes increase with increasing CS-factor. For all other subvolumes, the calculated volumes decrease with increasing CS-factor (Fig. 3a). Combining the measured relative volumes of all subjects, the observed differences between consecutive CS-factors were all significant (*p* < 0.05) for CS-factors ≥ 4 for all subvolumes (Tables 4, 5).

**Table 4:** FS-based volumes relative to CS-factor = 1, mean and SD across all subjects

CS-factor	4		8		12		16		20		32	
	Mean	SD	Mean	SD	Mean	SD	Mean	SD	Mean	SD	Mean	SD
BP	0.9997	0.0056	0.9956	0.0050	0.9849	0.0071	0.9770	0.0144	0.9683	0.0100	0.9133	0.0356
GM	0.9990	0.0035	0.9962	0.0062	0.9910	0.0064	0.9834	0.0127	0.9775	0.0082	0.9149	0.0248
WM	1.0004	0.0078	0.9954	0.0073	0.9790	0.0116	0.9677	0.0193	0.9551	0.0196	0.8993	0.0556
CSF	0.9657	0.0668	1.0128	0.0885	1.0886	0.1134	1.2417	0.1853	1.3358	0.1527	1.7707	0.3727

Relative volumes were calculated individually for each subject

*CS* compressed SENSE, *SD* standard deviation, *FS* FreeSurfer segmentation and volumetry tool, *BP* brain parenchyma, *WM* white matter, *GM* gray matter, *CSF* cerebrospinal fluid

**Table 5:** Comparison between FS-based relative volumes between consecutive CS-factors (CS = 1 vs. CS = 4, CS = 4 vs. CS = 8, etc.) across all subjects: *p*-values of paired *t* tests, Cohen’s *d* as a measure of effect size

CS-factors	1 vs. 4		4 vs. 8		8 vs. 12		12 vs. 16		16 vs. 20		20 vs. 32	
	*p*	*d*	*p*	*d*	*p*	*d*	*p*	*d*	*p*	*d*	*p*	*d*
BP	0.805	–	0.0031*	0.63	< 0.001*	2.46	0.0047*	0.60	0.0016*	0.68	< 0.001*	1.85
GM	0.159	–	0.0148*	0.50	< 0.001*	0.74	0.0043*	0.60	0.0129*	0.51	< 0.001*	2.41
WM	0.780	–	0.0279*	0.45	< 0.001*	2.14	< 0.001*	0.73	0.0013*	0.69	< 0.001*	1.41
CSF	0.013*	0.51	< 0.001*	− 0.54	0.001*	− 0.71	< 0.001*	− 0.90	0.0151*	− 0.50	< 0.001*	− 1.19

*CS* compressed SENSE, *FS* FreeSurfer segmentation and volumetry tool, *BP* brain parenchyma, *WM* white matter, *GM* gray matter, *CSF* cerebrospinal fluid

*Marks significant difference to volume at subsequent CS-factor (*p* < 0.05)

### Noise level analysis

To obtain a quantitative measure of image quality, we calculated NCR, which increases with more noise added to the images. Pearson correlation coefficient of the CS-factor and the NCR revealed a strong linear correlation (*R*^2^ = 0.98; see also Suppl. Fig. 1). NCR and the mean of the calculated relative volumes across subjects also showed strong linear correlations which were higher for MDB-based volumes (NCR vs. BP: *R*^2^ = 0.98, NCR vs. WM: *R*^2^ = 0.98, NCR vs. GM: *R*^2^ = 0.98, NCR vs. CSF: *R*^2^ = 0.98) than for FS-based volumes (NCR vs. BP: *R*^2^ = 0.81, NCR vs. WM: *R*^2^ = 0.80, NCR vs. GM: *R*^2^ = 0.94, NCR vs. CSF: *R*^2^ = 0.94), as visualized in Fig. 3b. This suggests that for MDB-based volumetry, the observed systematic CS-factor-dependent bias can be almost completely explained by the noise level increase induced by CS-acceleration. Analogously, for FS-based volumetry, the observed systematic CS-factor-dependent bias can, at least in large part, be explained by the noise level increase induced by CS-acceleration.

Additional analyses of smaller subcortical gray matter volumes of interest (pallidum, putamen, caudate and thalamus) resulted in higher CoV values compared to the analyzed whole-brain volumes BP, WM, GM and CSF (Supplementary Table 5).

### Reliability

Except for the FS-based measurements at CS-factor = 32, the narrow error bars in Fig. 2 give a good visual indication of the fairly low CoV. A CoV < 5% is considered acceptable [33]. Reliability assessed by ICC is considered to be poor (< 0.50), moderate (0.50–0.75), good (0.75–0.90) or excellent (> 0.90) [34].

#### MDB-based volumetry

For each CS-factor, the CoV of the repeatedly measured volumes was < 1% across all subjects and subvolumes (Table 6).^1^ Reliability assessed by ICC was good to excellent across all subvolumes up to CS-factor = 12 (Table 7).

**Table 6:** Coefficients of variation (CoV) in % of the MDB-based repeated volumetric measurements

CS-factor		1	4	8	12	16	20	32
BP	Subject 1	0.58	0.47	0.39	0.48	0.51	0.39	0.29
	Subject 2	0.13	0.64	0.48	0.55	0.47	0.49	0.47
	Subject 3	0.20	0.50	0.49	0.52	0.63	0.53	0.63
WM	Subject 1	0.12	0.40	0.37	0.30	0.44	0.43	0.20
	Subject 2	0.14	0.62	0.52	0.56	0.51	0.56	0.82
	Subject 3	0.33	0.56	0.43	0.54	0.69	0.60	0.54
GM	Subject 1	0.88	0.54	0.45	0.66	0.68	0.73	0.48
	Subject 2	0.22	0.74	0.52	0.58	0.55	0.50	0.32
	Subject 3	0.50	0.48	0.60	0.58	0.74	0.77	0.86
CSF	Subject 1	0.88	0.53	0.41	0.77	0.69	0.84	0.37
	Subject 2	0.29	0.54	0.43	0.42	0.49	0.43	0.37
	Subject 3	0.61	0.19	0.33	0.28	0.43	0.50	0.63

*CS* compressed SENSE, *MDB* md.brain segmentation and volumetry tool, *BP* brain parenchyma, *WM* white matter, *GM* gray matter, *CSF* cerebrospinal fluid

**Table 7:** Pairwise ICC to assess reliability between CS-factor = 1 and CS-factor = 4, 8, 12, 16, 20, 32, respectively, for MDB

MDB	ICC_1,4_	ICC_1,8_	ICC_1,12_	ICC_1,16_	ICC_1,20_	ICC_1,32_
BP	0.9150	0.8760	0.7691	0.7027	0.6429	0.5412
WM	0.9758	0.9395	0.9187	0.8936	0.8762	0.7945
GM	0.9212	0.8948	0.7628	0.6880	0.6048	0.4747
CSF	0.9855	0.9841	0.9312	0.8613	0.7922	0.6356

*ICC* intra-class correlation coefficient, *ICC*_*1,i*_ ICC between CS-factors 1 and *I*, *CS* compressed SENSE, *MDB* md.brain software tool, *BP* brain parenchyma, *WM* white matter, *GM* gray matter, *CSF* cerebrospinal fluid

#### FS-based volumetry

For each CS-factor < 32 and subvolumes BP, WM and GM, the CoV of the repeatedly measured volumes was < 1% across all subjects and subvolumes except for BP and GM at CS-factor = 16 in subject 2 (Table 8) ^1^. For CS-factor = 32, the repeatedly measured volumes showed significantly higher CoVs of up to 7% for subvolumes BP, WM and GM. For CSF, the CoVs were even higher and reached up to 16% for CS-factor < 32 and up to 25% for CS-factor = 32 (Table 7). Reliability assessed by ICC was good to excellent across all subvolumes up to CS-factor = 12 except for CSF which only showed a moderate reliability at CS-factor = 12 (Table 9).

**Table 8:** Coefficients of variation (CoV) in % of the FS-based repeated volumetric measurements

CS-factor		1	4	8	12	16	20	32
BP	Subject 1	0.30	0.64	0.46	0.76	0.65	0.69	4.45
	Subject 2	0.58	0.33	0.27	0.37	1.33	0.44	0.72
	Subject 3	0.31	0.46	0.41	0.71	0.52	0.77	2.44
WM	Subject 1	0.26	0.33	0.45	0.65	0.65	0.58	2.54
	Subject 2	0.17	0.21	0.36	0.38	0.90	0.53	1.44
	Subject 3	0.47	0.36	0.58	0.57	0.55	0.80	3.73
GM	Subject 1	0.34	0.97	0.68	0.95	0.83	0.88	6.75
	Subject 2	0.89	0.38	0.44	0.42	1.93	0.70	0.72
	Subject 3	0.83	0.67	0.70	0.99	0.60	0.91	1.74
CSF	Subject 1	7.80	8.15	4.76	8.68	10.53	11.77	24.47
	Subject 2	4.47	5.19	6.24	3.15	14.89	8.88	7.68
	Subject 3	3.58	5.30	9.67	9.36	7.09	4.15	14.45

*CS* compressed SENSE, *FS* FreeSurfer segmentation and volumetry tool, *BP* brain parenchyma, *WM* white matter, *GM* gray matter, *CSF* cerebrospinal fluid

**Table 9:** Pairwise ICC to assess reliability between CS-factor = 1 and CS-factor = 4, 8, 12, 16, 20, 32, respectively, for FS

FS	ICC_1,4_	ICC_1,8_	ICC_1,12_	ICC_1,16_	ICC_1,20_	ICC_1,32_
BP	0.9996	0.9994	0.9987	0.9932	0.9940	0.9187
WM	0.9998	0.9992	0.9992	0.9968	0.9980	0.9946
GM	0.9993	0.9986	0.9928	0.9797	0.9661	0.6894
CSF	0.9550	0.8501	0.7233	0.6369	0.7014	0.3418

*ICC* intra-class correlation coefficient, *ICC*_*1,i*_ ICC between CS-factors 1 and *I*, *CS* compressed SENSE, *FS* FreeSurfer software tool, *BP* brain parenchyma, *WM* white matter, *GM* gray matter, *CSF* cerebrospinal fluid

### Within-session and across-session reproducibility

Mean and SD of the estimated brain volumes within a single session and across sessions for MDB and FS, respectively, can be found in the supplementary material. The results indicate that, except for CS-factors > 16 and for the FS-based measurements of CSF, reproducibility is comparable when assessed across sessions as compared to within a single session.

## Discussion

In our study, we showed that MRI acquisition acceleration via Compressed SENSE (CS) has a systematic bias effect on volumetric brain measurements which correlate strongly with image noise levels. This bias effect alters tissue quantification depending on the software used and analyzed subvolume. The present study is among the first to link a systematic volumetric bias of brain tissue with state of the art MR image acceleration techniques. The precision of the volumetric brain measurements remains high, even at a relatively high degree of acceleration.

For both software tools used, MDB and FS, we showed a systematic bias of the volumetric brain measurements proportional to the employed CS-factor. For both software tools and all analyzed subvolumes, this bias shows a strong linear behavior what was confirmed by linear correlation analysis (*R* > 0.95, *p* < 0.01). This translates into increasing WM volumes for MDB and increasing CSF volumes for FS (Fig. 3). Considerable differences in intracranial tissue segmentation between SPM/CAT12 and FS are well known and have been demonstrated before. In particular, pronounced differences regarding inter-method and intra-method segmentations [35] as well as a dependence of inter-method variations in calculated volumes on the analyzed brain compartment [36] have been reported. In a recent study by Palumbo et al., the authors showed a systematic oversegmentation of GM volume by CAT12 compared to FS [37]. This systematic difference in tissue classification between voxel-based morphometry and surface-based morphometry may be the reason for differences in absolute tissue volumes between MDB and FS.

Previous studies on parallel imaging techniques and brain volumes did not find a corresponding relationship investigating brain volumetry in healthy adults and dementia patients [38, 39]. However, these previous studies only investigated the effect of PI on volumetric brain measurements without additional compressed sensing. In our study, imaging data acquisition and processing was performed with NBV as the intended clinical application, i.e. for comparison of brain volumes with a normal cohort. For the rating of atrophy in dementia patients, providing additional quantitative information, for example, in the form of deviation maps or volume percentile curves, was shown to have a beneficial effect on diagnostic accuracy [4, 5]. Although precise percentage cut-off values have not been defined, volumes below the fifth percentile or below two standard deviations are usually considered pathologic [4, 5]. Knowing about systematic, CS-dependent tissue classification could lead to a more individual adjustment of these conventions for pathologic tissue atrophy, e.g. by means of a scaling factor. Since we demonstrated that the found systematic bias can vary across brain regions and the utilized analysis software, it may be reasonable to derive specific scaling factors depending on the respective setting. The alternative approach of matching CS-factors within the control cohort to the patient undergoing NBV evaluation seems not feasible since these cohorts are usually fixed.

NCR was calculated as quantitative measure of image quality and increased with the degree of acceleration as evidenced by the strong linear correlation with CS-factor. NCR and the calculated relative volumes also showed a strong linear correlation. This indicates that for MDB-based volumetry, and almost to the same extent for FS-based volumetry, the observed systematic bias is driven by the noise level increase induced by CS-acceleration. Furthermore, the enlarged views in Fig. 1 suggest that noise is more pronounced towards the image center (Fig. 1). Analyzing smaller subcortical regions of interest, we found less reliable results compared to whole-brain volumes BP, WM, GM and CSF. This supports the hypothesis of a non-uniform noise distribution with increased noise levels in the central regions. Previous studies about the relationship between image noise and measured brain volumes are scarce. In a reliability study of MRI measurements, Maclaren et al. [40] did not find any correlation between lateral ventricle volumes and image noise. However, the range of observed image noise was considerably narrower than in the previous study since the used sequences were not modified along the repeated measurements. The increased NCR is most likely the primary cause of the found CS-dependent systematic bias. Therefore, future investigations applying different denoising strategies to the acquired MRI data prior to volumetric measurements could be a way to potentially reduce the biasing effect.

Except for FS-based volumetry of CSF, we have shown acceptable intra-subject reliability of the measured brain volumes using two different software tools. Interestingly, this holds true even for high acceleration factors up to CS-factor = 20. In 2009, Lindholm et al. analyzed intra-subject reliability of brain volumetry accelerated by the PI technique GRAPPA. The reported CoVs for GM and WM have a comparable range (0.4–1.6%) as the values found in our study [38]. One potential explanation for the preserved precision of measured brain volumes could be that the shortened acquisition time and associated reduction of motion artifacts compensates for the increased noise level at higher CS-factors. The low intrinsic measurement variability of CS-accelerated volumetric brain measurements found in our study can be considered a prerequisite for its application. Of note, no pre-processing for longitudinal brain volume evaluation was performed, since we focused on a single timepoint NBV scenario. This means that the observed CoV could potentially be reduced through optimized preprocessing steps for longitudinal brain volume assessment.

The present study is not without limitations. First, we only included three young healthy subjects. To validate our results in clinically relevant patient groups, further studies with a higher number of subjects, a broader age range and subjects suffering from neurodegenerative diseases are necessary. Additionally, these patients might be more prone to motion artifacts. Second, our study was primarily designed for the analysis of CS-acceleration in the context of NBV and not for the assessment of volume changes over time. To evaluate the impact on longitudinal brain volume analysis, a different study design and data processing would be needed. Third, for certain subvolumes, the found systematic bias shows marked differences between MDB and FS which cannot be explained in detail. To better comprehend the mechanism behind these differences, further investigations into the algorithms of the two software tools would be required but were beyond the scope of this study. Finally, the noise distribution of the imaging data was only assessed by visual inspection and the analysis of smaller subcortical volumes of interest in comparison to whole-brain volumes. Future studies should consider performing additional measurements on a uniform phantom image to improve the spatial characterization of image noise with regard to increasing acceleration factors.

## Conclusion

We found that CS-accelerated MRI poses a systematic bias on measured brain volumes with differential effects on tissue classes depending on the volumetry pipeline used, at mostly preserved measurement precision. This bias effect leading to impaired accuracies of volume estimations is mostly explained by increasing image noise and should be taken into account when comparing brain volumes with external databases.

## Supplementary Information

Below is the link to the electronic supplementary material.

Supplementary file1 (DOCX 2966 kb)

## Abbreviations

AP: Anterior–posterior direction
BP: Brain parenchyma (combined gray and white matter)
CoV: Coefficient of variation
CS: Compressed SENSE
CS-factor: Compressed SENSE reduction factor
CSF: Cerebrospinal fluid
FH: Feet-head direction
FS: FreeSurfer segmentation and volumetry tool
GM: Gray matter
GRAPPA: Generalized autocalibrating partially parallel acquisition
ICC: Intraclass correlation coefficient
ICC_1,__*i*_: ICC between CS-factors 1 and *i*
IRB: Institutional review board
MDB: Md.brain segmentation and volumetry tool
ml: Milliliter
MRI: Magnetic resonance imaging
NBV: Normative brain volumetry
NCR: Noise-to-contrast-ratio
PI: Parallel imaging
R: Pearson correlation coefficient
RL: Right-left direction
SD: Standard deviation
SENSE: Sensitivity encoding
SPM: Statistical parametric mapping
T: Tesla
T1w: T1-weighted
TE: Time of echo
TFE: Turbo field echo
TR: Time of repetition
VBM: Voxel-based morphometry
WM: White matter

## Footnotes

1 One of the nine measurements at CS-factor = 16 in subject 2 was detected as an outlier and excluded from the calculation of the CoV.
