# Supplementary material for: Effect of MRI acquisition acceleration via compressed sensing and parallel imaging on brain volumetry
Source: MAGMA. 2021 Jan 27;34(4):487–97. doi: 10.1007/s10334-020-00906-9 (PMC8338844; doi:10.1007/s10334-020-00906-9)
Supplement: Supplementary file 1 — Supplementary file1 (DOCX 2966 kb) [file 10334_2020_906_MOESM1_ESM.docx]

**Supplementary Material**

| **CS-factor** |  | **4** |  | **8** |  | **12** |  | **16** |  | **20** |  | **32** |  |
| --- | --- | --- | --- | --- | --- | --- | --- | --- | --- | --- | --- | --- | --- |
|  |  | **Mean** | **SD** | **Mean** | **SD** | **Mean** | **SD** | **Mean** | **SD** | **Mean** | **SD** | **Mean** | **SD** |
| **Subject 1** | **BP** | 1086.83 | 1.76 | 1080.69 | 3.03 | 1052.85 | 6.60 | 1029.68 | 5.80 | 1014.56 | 6.07 | 963.84 | 1.37 |
|  | **WM** | 409.52 | 0.68 | 413.71 | 0.39 | 414.78 | 0.77 | 419.35 | 2.47 | 423.68 | 2.61 | 433.21 | 0.83 |
|  | **GM** | 677.31 | 1.11 | 666.98 | 2.67 | 638.07 | 5.84 | 610.34 | 3.78 | 590.88 | 5.55 | 530.63 | 0.77 |
|  | **CSF** | 762.87 | 2.05 | 753.27 | 2.46 | 721.55 | 7.94 | 690.05 | 3.80 | 668.92 | 6.14 | 607.82 | 1.93 |
| **Subject 2** | **BP** | 1128.72 | 4.25 | 1133.51 | 3.98 | 1121.27 | 2.02 | 1115.69 | 3.90 | 1112.98 | 1.51 | 1095.27 | 2.62 |
|  | **WM** | 437.88 | 1.16 | 443.87 | 2.04 | 445.69 | 0.83 | 453.53 | 2.19 | 460.86 | 0.52 | 475.28 | 1.98 |
|  | **GM** | 690.84 | 3.11 | 689.64 | 1.96 | 675.58 | 2.16 | 662.16 | 3.20 | 652.13 | 1.36 | 619.99 | 1.24 |
|  | **CSF** | 765.30 | 1.71 | 760.00 | 1.18 | 744.63 | 3.08 | 729.08 | 3.52 | 719.21 | 2.21 | 688.86 | 1.48 |
| **Subject 3** | **BP** | 1167.83 | 2.40 | 1170.41 | 4.73 | 1163.97 | 4.63 | 1157.45 | 3.06 | 1143.89 | 3.39 | 1112.81 | 2.18 |
|  | **WM** | 427.91 | 0.79 | 435.81 | 1.01 | 440.21 | 0.66 | 447.69 | 0.74 | 450.46 | 4.33 | 466.01 | 2.70 |
|  | **GM** | 739.93 | 1.76 | 734.60 | 4.72 | 723.75 | 4.15 | 709.77 | 3.65 | 693.43 | 5.47 | 646.80 | 2.16 |
|  | **CSF** | 656.80 | 0.32 | 648.82 | 3.17 | 638.24 | 2.81 | 625.41 | 2.08 | 612.72 | 3.83 | 577.88 | 3.32 |

**Supplementary Table 1**

Mean and standard deviation (SD) of the **MDB**-based repeated volumetric measurements (in ml) **within a single session** (3 repeated scans per session); SD, standard deviation; MDB, md.brain segmentation and volumetry tool; ml, milliliter; BP, brain parenchyma; WM, white matter; GM, gray matter; CSF, cerebrospinal fluid.

| **CS-factor** | | **4** | | **8** | | **12** | | **16** | | **20** | | **32** | |
| --- | --- | --- | --- | --- | --- | --- | --- | --- | --- | --- | --- | --- | --- |
|  |  | **Mean** | **SD** | **Mean** | **SD** | **Mean** | **SD** | **Mean** | **SD** | **Mean** | **SD** | **Mean** | **SD** |
| **Subject 1** | **BP** | 1087.46 | 3.70 | 1082.43 | 3.26 | 1054.90 | 2.20 | 1031.88 | 2.15 | 1013.68 | 1.05 | 964.20 | 2.67 |
|  | **WM** | 409.78 | 1.24 | 413.67 | 1.42 | 414.97 | 0.31 | 418.88 | 0.68 | 423.70 | 0.32 | 432.93 | 0.25 |
|  | **GM** | 677.69 | 2.47 | 668.76 | 2.13 | 639.93 | 2.23 | 613.01 | 2.78 | 589.98 | 1.30 | 531.27 | 2.66 |
|  | **CSF** | 763.75 | 3.32 | 755.24 | 2.31 | 722.91 | 1.84 | 692.13 | 2.28 | 666.75 | 2.02 | 607.01 | 2.04 |
| **Subject 2** | **BP** | 1128.72 | 7.03 | 1132.40 | 4.64 | 1124.27 | 6.61 | 1113.06 | 2.32 | 1113.89 | 5.98 | 1096.04 | 5.42 |
|  | **WM** | 436.38 | 2.25 | 443.54 | 0.90 | 446.97 | 2.48 | 453.22 | 1.76 | 461.00 | 2.66 | 476.15 | 4.29 |
|  | **GM** | 692.34 | 5.36 | 688.86 | 3.75 | 677.29 | 4.14 | 659.84 | 2.96 | 652.89 | 3.33 | 619.89 | 1.16 |
|  | **CSF** | 765.38 | 4.29 | 757.49 | 3.51 | 744.56 | 2.79 | 726.80 | 2.06 | 718.18 | 2.73 | 686.85 | 1.94 |
| **Subject 3** | **BP** | 1166.13 | 6.44 | 1169.16 | 5.93 | 1162.35 | 6.37 | 1153.88 | 7.51 | 1144.40 | 5.62 | 1112.61 | 7.94 |
|  | **WM** | 426.92 | 2.68 | 435.07 | 1.89 | 438.40 | 2.67 | 444.53 | 3.20 | 450.67 | 1.01 | 466.67 | 2.06 |
|  | **GM** | 739.22 | 3.78 | 734.09 | 4.08 | 723.95 | 4.06 | 709.36 | 5.20 | 693.73 | 4.62 | 645.94 | 6.01 |
|  | **CSF** | 655.77 | 0.90 | 648.22 | 1.17 | 638.61 | 1.14 | 625.40 | 2.27 | 612.53 | 1.99 | 576.80 | 3.59 |

**Supplementary Table 2**

Mean and SD of the **MDB**-based repeated volumetric measurements (in ml) **across sessions** (3 sessions; for each subject, the 3 scans per session were averaged); SD, standard deviation; MDB, md.brain segmentation and volumetry tool; ml, milliliter; BP, brain parenchyma; WM, white matter; GM, gray matter; CSF, cerebrospinal fluid.

| **CS-factor** | | **4** | | **8** | | **12** | | **16** | | **20** | | **32** | |
| --- | --- | --- | --- | --- | --- | --- | --- | --- | --- | --- | --- | --- | --- |
|  |  | **Mean** | **SD** | **Mean** | **SD** | **Mean** | **SD** | **Mean** | **SD** | **Mean** | **SD** | **Mean** | **SD** |
| **Subject 1** | **BP** | 1323.73 | 10.25 | 1317.73 | 4.40 | 1296.67 | 10.83 | 1284.98 | 10.01 | 1272.97 | 10.29 | 1120.87* | 69.54* |
|  | **WM** | 547.87 | 1.64 | 545.64 | 3.26 | 546.17 | 3.52 | 540.47 | 5.85 | 539.49 | 3.05 | 509.93* | 12.85* |
|  | **GM** | 746.07 | 9.05 | 740.65 | 3.84 | 721.55 | 6.15 | 710.21 | 5.55 | 698.82 | 7.02 | 590.33* | 63.32* |
|  | **CSF** | 629.13 | 56.84 | 686.97 | 19.36 | 808.37 | 57.28 | 884.40 | 84.78 | 960.50 | 55.09 | 922.95* | 156.77* |
| **Subject 2** | **BP** | 1327.42 | 5.14 | 1323.78 | 3.32 | 1307.92 | 3.70 | 1304.16 | 6.32 | 1292.45 | 6.15 | 1248.64 | 10.88 |
|  | **WM** | 567.89 | 1.66 | 565.54 | 3.11 | 562.45 | 1.96 | 559.50 | 1.60 | 553.07 | 3.83 | 524.53 | 10.12 |
|  | **GM** | 720.04 | 2.32 | 720.37 | 2.70 | 706.32 | 1.18 | 705.77 | 5.26 | 697.92 | 6.22 | 679.14 | 2.46 |
|  | **CSF** | 797.00 | 52.03 | 885.63 | 82.72 | 902.23 | 5.74 | 1180.63 | 115.68 | 1138.00 | 158.64 | 1795.67 | 97.64 |
| **Subject 3** | **BP** | 1077.02 | 3.07 | 1079.89 | 3.04 | 1070.35 | 4.18 | 1066.45 | 4.77 | 1057.31 | 4.68 | 997.36* | 18.44* |
|  | **WM** | 442.32 | 0.51 | 441.29 | 3.82 | 436.57 | 3.52 | 436.56 | 2.72 | 430.05 | 2.60 | 405.05* | 9.21* |
|  | **GM** | 610.64 | 3.63 | 611.87 | 1.58 | 607.79 | 1.72 | 601.06 | 2.42 | 594.30 | 2.88 | 557.55* | 9.00* |
|  | **CSF** | 696.77 | 33.89 | 708.70 | 63.87 | 791.67 | 34.97 | 848.53 | 53.19 | 953.50 | 39.18 | 1117.27* | 136.43* |

**Supplementary Table 3**

Mean and SD of the **FS**-based repeated volumetric measurements (in ml) **within a single session** (3 repeated scans per session, * mark sessions with only 2 repeated scans per session due to failure of the FS segmentation algorithm); SD, standard deviation; FS, FreeSurfer segmentation and volumetry tool; ml, milliliter; BP, brain parenchyma; WM, white matter; GM, gray matter; CSF, cerebrospinal fluid.

| **CS-factor** | | **4** | | **8** | | **12** | | **16** | | **20** | | **32** | |
| --- | --- | --- | --- | --- | --- | --- | --- | --- | --- | --- | --- | --- | --- |
|  |  | **Mean** | **SD** | **Mean** | **SD** | **Mean** | **SD** | **Mean** | **SD** | **Mean** | **SD** | **Mean** | **SD** |
| **Subject 1** | **BP** | 1330.21 | 5.72 | 1322.31 | 5.41 | 1306.36 | 8.73 | 1290.95 | 5.80 | 1275.03 | 3.93 | 1152.32 | 33.29 |
|  | **WM** | 548.00 | 1.76 | 546.86 | 1.71 | 546.16 | 2.98 | 541.02 | 0.77 | 539.71 | 1.78 | 497.47 | 11.15 |
|  | **GM** | 751.10 | 4.35 | 744.17 | 3.86 | 728.16 | 5.74 | 715.64 | 5.32 | 699.22 | 2.53 | 610.62 | 18.66 |
|  | **CSF** | 627.47 | 24.58 | 686.56 | 12.79 | 763.21 | 43.42 | 843.78 | 41.28 | 885.53 | 73.06 | 1227.85 | 268.22 |
| **Subject 2** | **BP** | 1329.86 | 2.70 | 1325.70 | 2.49 | 1312.53 | 4.88 | 1296.43 | 17.86 | 1295.23 | 2.65 | 1249.65 | 1.09 |
|  | **WM** | 568.25 | 0.81 | 564.89 | 0.68 | 562.39 | 1.37 | 555.16 | 6.18 | 553.31 | 0.99 | 527.39 | 2.81 |
|  | **GM** | 721.85 | 1.87 | 721.59 | 2.35 | 708.96 | 3.15 | 698.90 | 11.91 | 699.70 | 2.06 | 678.29 | 3.33 |
|  | **CSF** | 833.37 | 33.01 | 871.54 | 14.32 | 924.69 | 29.13 | 1114.84 | 69.59 | 1194.83 | 67.86 | 1703.12 | 81.81 |
| **Subject 3** | **BP** | 1080.26 | 5.33 | 1076.65 | 3.59 | 1065.76 | 4.25 | 1065.53 | 0.93 | 1051.21 | 5.31 | 982.16 | 22.07 |
|  | **WM** | 440.81 | 1.31 | 440.58 | 1.61 | 436.26 | 0.68 | 435.85 | 0.89 | 430.59 | 3.08 | 398.93 | 12.35 |
|  | **GM** | 613.25 | 4.10 | 610.00 | 4.23 | 603.54 | 3.75 | 601.43 | 0.64 | 591.09 | 2.78 | 551.10 | 9.24 |
|  | **CSF** | 718.18 | 33.51 | 721.63 | 63.59 | 757.11 | 41.39 | 839.30 | 16.02 | 933.34 | 19.04 | 1084.47 | 57.35 |

**Supplementary Table 4**

Mean and SD of the **FS**-based repeated volumetric measurements (in ml) **across sessions** (3 sessions; for each subject, the 3 scans per session were averaged); SD, standard deviation; FS, FreeSurfer segmentation and volumetry tool; ml, milliliter; BP, brain parenchyma; WM, white matter; GM, gray matter; CSF, cerebrospinal fluid.

| **CS-factor** | | **1** | **4** | **8** | **12** | **16** | **20** | **32** |
| --- | --- | --- | --- | --- | --- | --- | --- | --- |
| **Pallidum** | **Subject 1** | 1.32 | 1.48 | 1.28 | 0.88 | 5.98 | 6.03 | 6.86 |
|  | **Subject 2** | 0.86 | 1.86 | 1.26 | 2.28 | 5.41 | 3.75 | 4.54 |
|  | **Subject 3** | 1.69 | 1.30 | 1.19 | 1.71 | 2.67 | 5.37 | 5.51 |
| **Putamen** | **Subject 1** | 1.93 | 4.36 | 3.85 | 4.61 | 15.62 | 14.50 | 69.53 |
|  | **Subject 2** | 4.18 | 4.46 | 4.61 | 6.55 | 12.95 | 7.80 | 11.43 |
|  | **Subject 3** | 3.13 | 1.85 | 2.43 | 3.14 | 14.62 | 16.27 | 16.59 |
| **Caudate** | **Subject 1** | 2.08 | 1.46 | 1.21 | 1.28 | 2.62 | 2.30 | 4.99 |
|  | **Subject 2** | 0.31 | 1.06 | 0.89 | 1.14 | 2.90 | 2.87 | 7.23 |
|  | **Subject 3** | 1.12 | 0.79 | 0.87 | 0.70 | 1.08 | 1.73 | 12.05 |
| **Thalamus** | **Subject 1** | 3.39 | 3.12 | 2.89 | 3.10 | 10.06 | 14.39 | 23.37 |
|  | **Subject 2** | 5.44 | 2.25 | 6.69 | 5.23 | 10.13 | 4.43 | 7.49 |
|  | **Subject 3** | 1.01 | 2.50 | 2.78 | 3.95 | 6.08 | 9.72 | 8.00 |

**Supplementary Table 5:** CoV in % of the FS-based repeated volumetric measurements for 4 smaller segmented subcortical gray matter regions; CoV, Coefficient of variation; FS, FreeSurfer segmentation and volumetry tool.

*
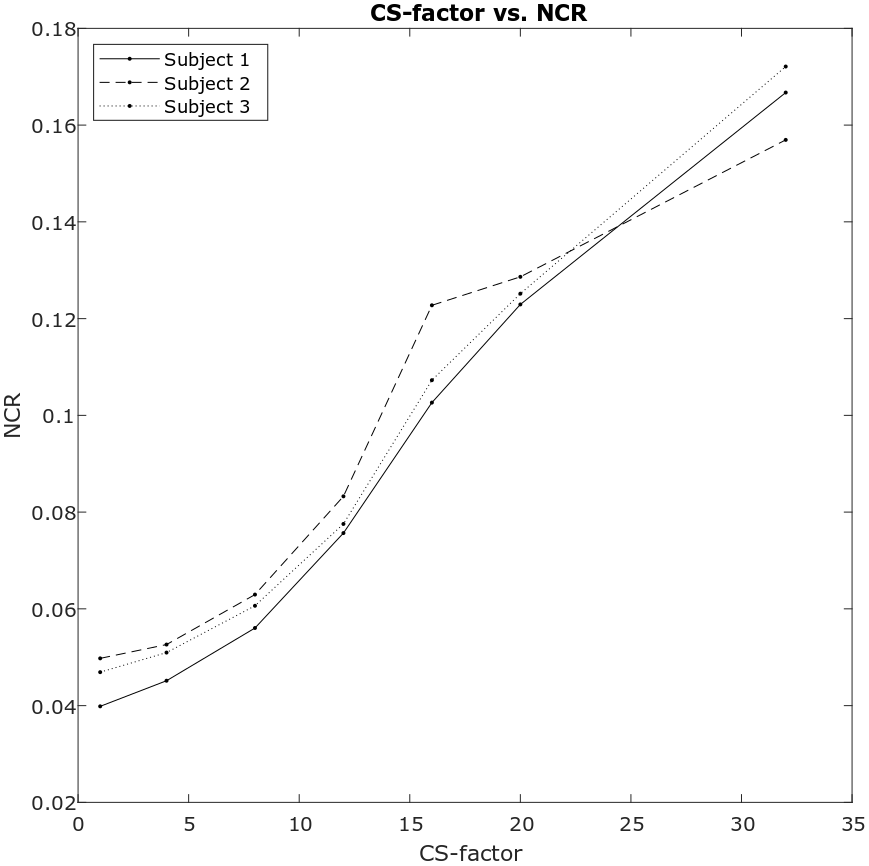
*

**Supplementary Figure 1**

CS-factor vs. NCR for each subject; CS, Compressed Sense, NCR, noise-to-contrast ratio.

**
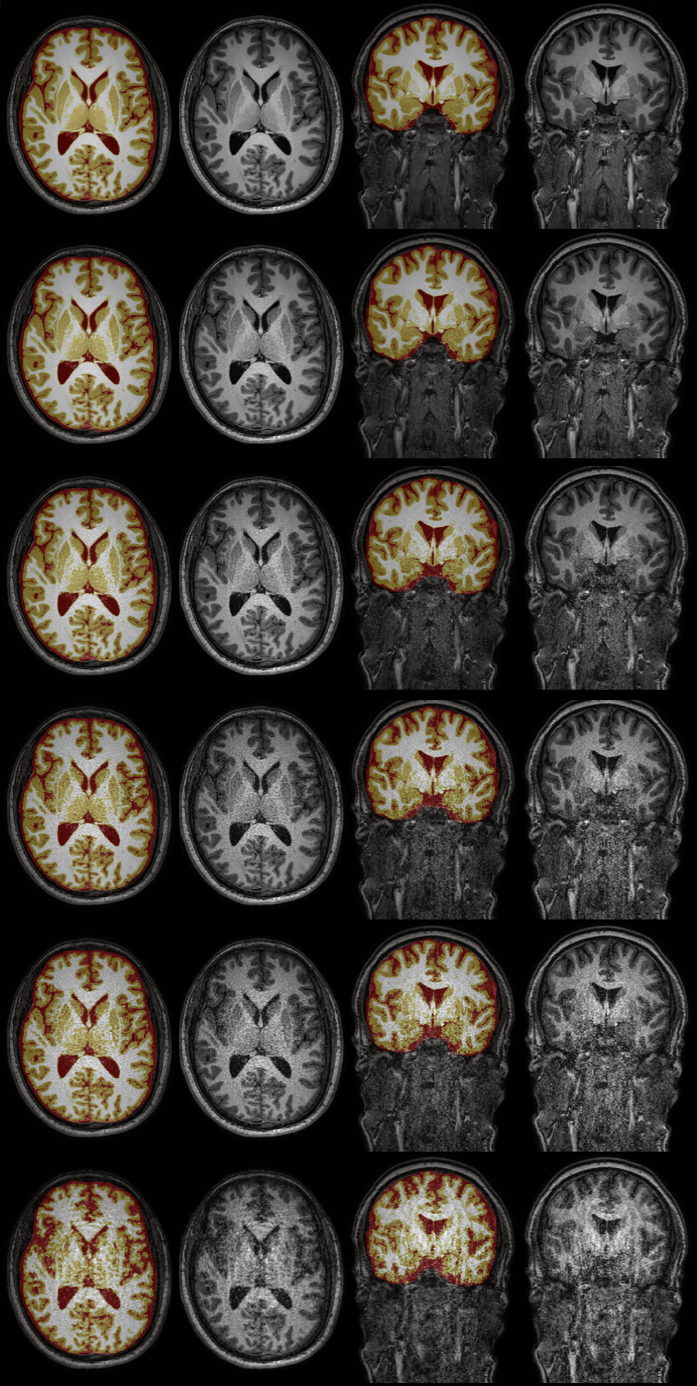
**

**Supplementary Figure 2a**

Sample axial and coronal reconstructions of the acquired 3D-T1-weighted brain images at increasing CS-factors (CS-factor = 4, 8, 12, 16, 20, and 32 from top to bottom row) of one of the subjects with color-coded overlays of the brain regions segmented by MDB: WM (white), GM (yellow) and CSF (red); CS, Compressed Sense; MDB, md.brain software tool; WM, white matter; GM, gray matter; CSF, cerebrospinal fluid.


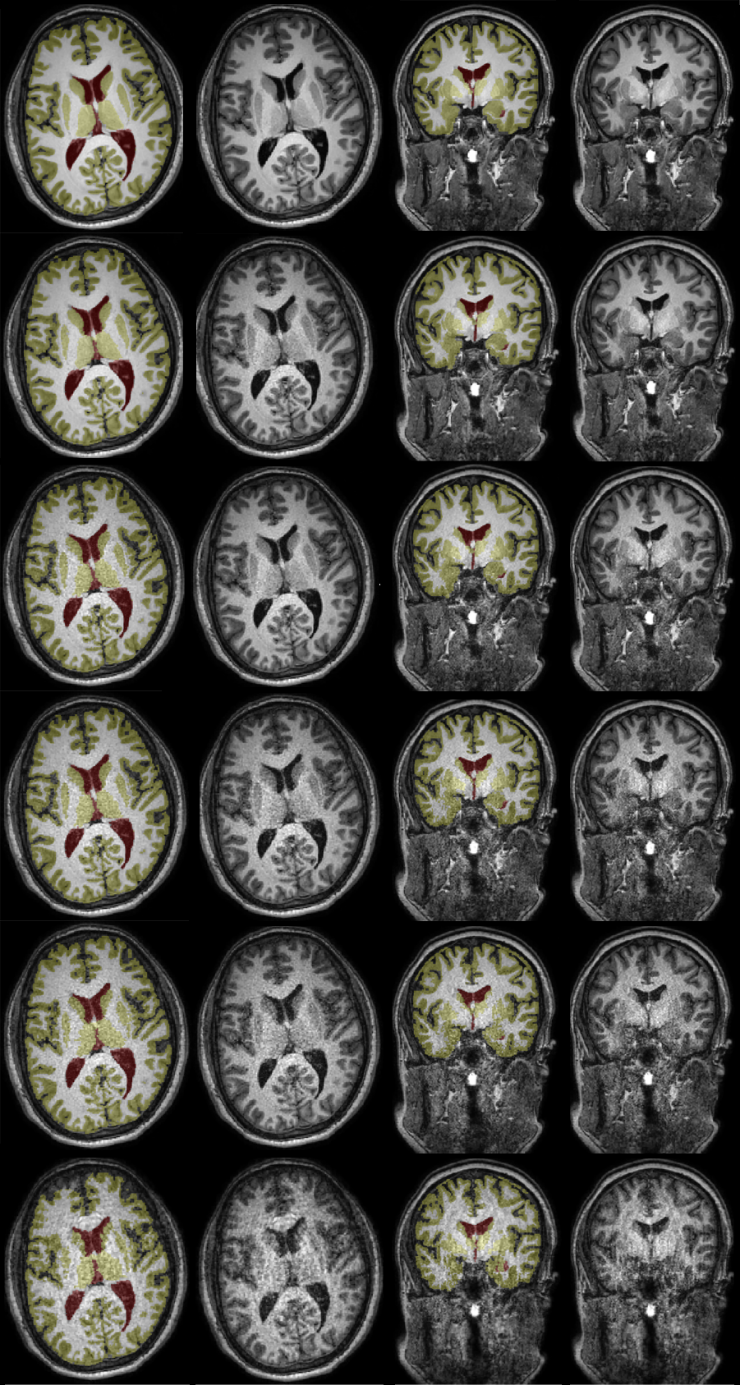


**Supplementary Figure 2b**

Sample axial and coronal reconstructions of the acquired 3D-T1-weighted brain images at increasing CS-factors (CS-factor = 4, 8, 12, 16, 20, and 32 from top to bottom row) of one of the subjects with color-coded overlays of the brain regions segmented by FS: WM (white), GM (yellow) and CSF (red); CS, Compressed Sense FS; FreeSurfer software tool; WM, white matter; GM, gray matter; CSF, cerebrospinal fluid.
